# The genomic environment around the Aromatase gene: evolutionary insights

**DOI:** 10.1186/1471-2148-5-43

**Published:** 2005-08-12

**Authors:** L Filipe C Castro, Miguel M Santos, Maria A Reis-Henriques

**Affiliations:** 1 CIIMAR – Centre of Marine and Environmental Research, Rua dos Bragas 289, 4050-123, Oporto, Portugal; 2 ICBAS – Institute of Biomedical Sciences Abel Salazar, Largo Professor Abel Salazar, 2, 4099-003, Oporto, Portugal

## Abstract

**Background:**

The cytochrome P450 aromatase (*CYP19*), catalyses the aromatisation of androgens to estrogens, a key mechanism in vertebrate reproductive physiology. A current evolutionary hypothesis suggests that *CYP19 *gene arose at the origin of vertebrates, given that it has not been found outside this clade. The human *CYP19 *gene is located in one of the proposed MHC-paralogon regions (HSA15q). At present it is unclear whether this genomic location is ancestral (which would suggest an invertebrate origin for *CYP19*) or derived (genomic location with no evolutionary meaning). The distinction between these possibilities should help to clarify the timing of the *CYP19 *emergence and which *taxa *should be investigated.

**Results:**

Here we determine the "genomic environment" around *CYP19 *in three vertebrate species *Homo sapiens*, *Tetraodon nigroviridis *and *Xenopus tropicalis*. Paralogy studies and phylogenetic analysis of six gene families suggests that the *CYP19 *gene region was structured through "en bloc" genomic duplication (as part of the MHC-paralogon formation). Four gene families have specifically duplicated in the vertebrate lineage. Moreover, the mapping location of the different paralogues is consistent with a model of "en bloc" duplication. Furthermore, we also determine that this region has retained the same gene content since the divergence of Actinopterygii and Tetrapods. A single inversion in gene order has taken place, probably in the mammalian lineage. Finally, we describe the first invertebrate *CYP19 *sequence, from *Branchiostoma floridae*.

**Conclusion:**

Contrary to previous suggestions, our data indicates an invertebrate origin for the aromatase gene, given the striking conservation pattern in both gene order and gene content, and the presence of aromatase in amphioxus. We propose that *CYP19 *duplicated in the vertebrate lineage to yield four paralogues, followed by the subsequent loss of all but one gene in vertebrate evolution. Finally, we suggest that agnathans and lophotrocozoan protostomes should be investigated for the presence of aromatase.

## Background

The cytochrome P450 aromatase (*CYP19*) is a member of a large superfamily of enzymes named cytochrome P450, which are involved in many physiological functions, such as steroid biosynthesis [1]. *CYP19 *is a steroidogenic enzyme which catalyses the aromatisation of androgens to estrogens. Thus, aromatase activity is essential for maintaining a physiological balance between androgens and estrogens, a critical aspect in the reproductive function of vertebrates; in humans, a P450 aromatase mutation leads to sterility [2]. For many other vertebrate groups, it as been demonstrated that *CYP19 *plays a key role in sex differentiation [3].

While several CYP450 genes are universally distributed, *CYP19 *is so far restricted to the vertebrate lineage. In mammals [4], birds [5], amphibians [6], reptiles [7] and cartilaginous fishes [8], a single gene has been isolated. In most Actinopterygii, however, two genes, *Cyp19a *and *Cyp19b*, encode two different transcripts expressed in the ovary and brain respectively [9]. Linkage data from zebrafish clearly suggests that these two genes are most likely the result of a genome duplication in the ray-finned bony fish lineage [9]. Despite intensive research, the ancestry of *CYP19 *genes is yet to be deciphered. No orthologue has been described from fully sequenced invertebrate genomes, like *Drosophila melanogaster*, *Ciona intestinalis *or *Caenorhabditis elegans *[10]. Thus, it has been suggested that the *CYP19 *gene arose at the origin of vertebrates [10,11]. Nevertheless, there is now strong evidence indicating that these model invertebrate species have experienced extensive gene loss [12]. Significantly, the estrogen receptor which was thought to have emerged in vertebrate ancestry, has now been documented in the lophotrocozoan protostome *Aplysia californica *[13].

**Figure 1:**
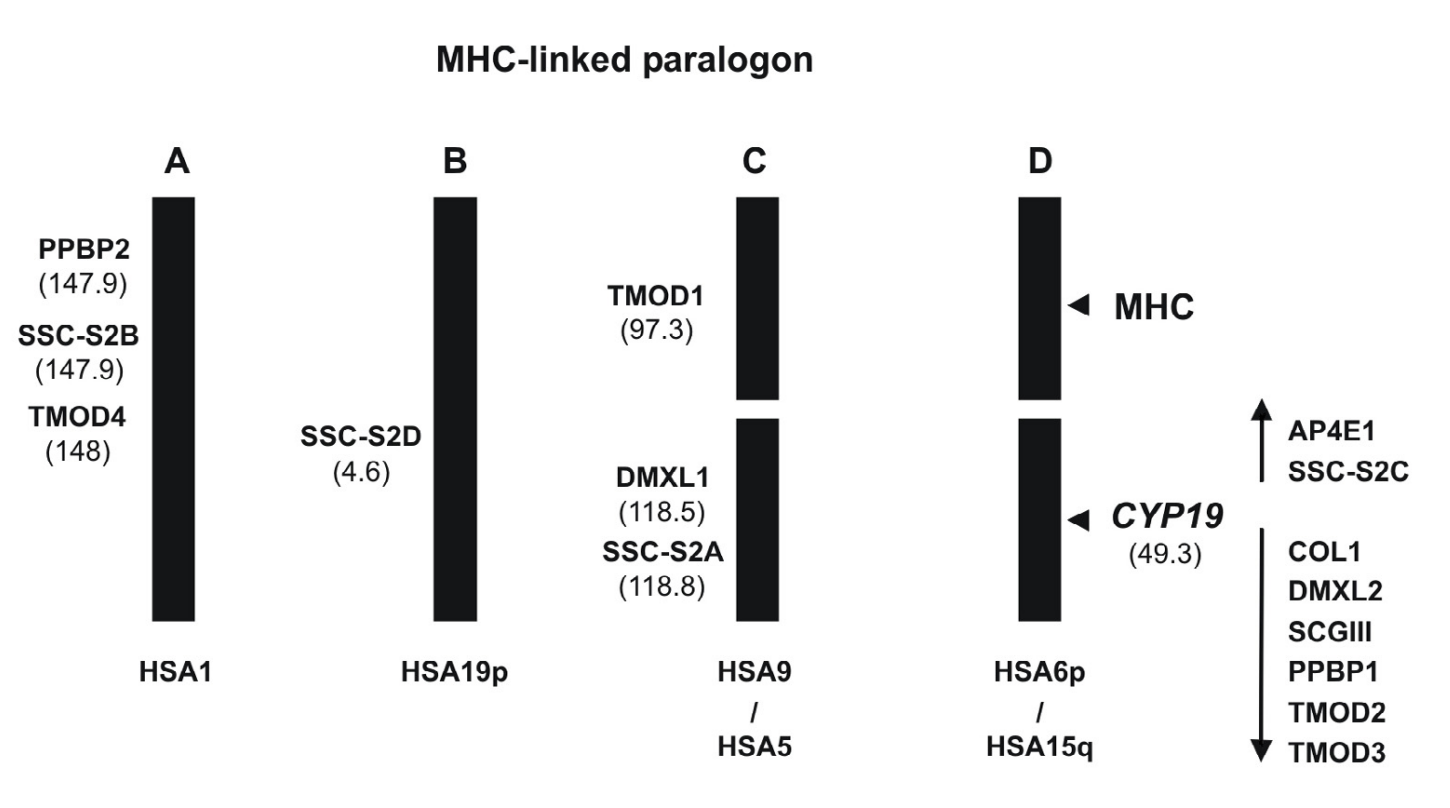
The MHC-paralogon. Two sets of paralogy regions are intact (A and B) while the remaining 2 are broken (C and D) (adapted from [20]). The map location of the MHC, CYP19 and the surrounding genes (and paralogues) is shown. In parentheses the genomic distance in megabases to the p telomere.

Paralogy regions (or paralogons) consist of a series of linked genes (unrelated) on one chromosome, many of which have linked homologues (or paralogues) on at least another chromosome (typically four) [14,15]. Two main scenarios have been put forward to account for their presence: evolutionary remnants of chromosomal "en bloc" duplications or genome duplication, followed by gene loss and inversions [16,17]; *or *they reflect independent tandem duplications of each gene family followed by adaptive groupings of genes on different chromosomes [18]. The term "en bloc" duplication is used here in the context described by Abi-Rached et al. [17]. One of the best characterised examples of a paralogon includes the genes around the MHC complex on human chromosome 6, with homologues on chromosomes 1, 9, 5, 15 and 19 [19,20] (figure 1). Despite other views [18], the most recent findings indicate that an ancestral MHC-like region/chromosome duplicated "en bloc" twice in early vertebrate ancestry to yield a four-array paralogon (figure 1) [17,19-22]. Accordingly, vertebrate genes within these regions should have multiple copies (up to four) equally related to single invertebrate orthologues [23]. However, this is not the case for a substantial proportion of gene families. Gene loss, genomic rearrangements and insertions in both vertebrate and invertebrate genomes will obscure the correct evolutionary pattern of gene families within paralogons [23]. This is particularly evident for gene families which are vertebrate single copy. If a vertebrate one-member gene family maps to a paralogon and no orthologue is found in invertebrate model species what should we conclude regarding the ancestry of such a gene family? The human *CYP19 *gene follows this pattern. It maps to HSA15q, a region proposed to be part of the MHC-paralogon [20] (figure 1). This genomic location is highly suggestive for a invertebrate origin of *CYP19*. Nevertheless, since no paralogues are found in other chromosomal regions, it could well be the case of a genomic location with no evolutionary meaning.

Phylogenetics, paralogy and comparative genomics can be a particularly powerful tool to address issues of gene ancestry. Here, we analysed the evolutionary history of the genes in close physical proximity to the aromatase gene(s) in several vertebrate species (*Homo sapiens*, *Tetraodon nigroviridis *and *Xenopus tropicalis*). Through phylogenetic analysis we demonstrate that the *CYP19 *region was structured most likely by "en bloc" genomic duplication (as part of the MHC-paralogon). Most importantly, we also determine that this region has retained the same gene content and overall organisation (*CYP19 *included) without any gene insertion in the three lineages. A single inversion of gene order has occurred in the mammalian clade. Finally, we describe for the first time an invertebrate *CYP19 *partial sequence from *Branchiostoma floridae*. We propose that the aromatase gene family is much older than previously hinted.

## Results and discussion

In this study, we sequentially addressed three questions. First, we determined the duplication pattern (pre or post vertebrate radiation) of the gene families in close proximity to the human *CYP19*. A further test analysed the ancestry of the human aromatase genomic location (ancestral *versus *derived). Finally, we investigated the presence of *CYP19 *in other invertebrate species (*B. floridae*), other than those previously explored.

### Phylogeny and paralogy

**Figure 2:**
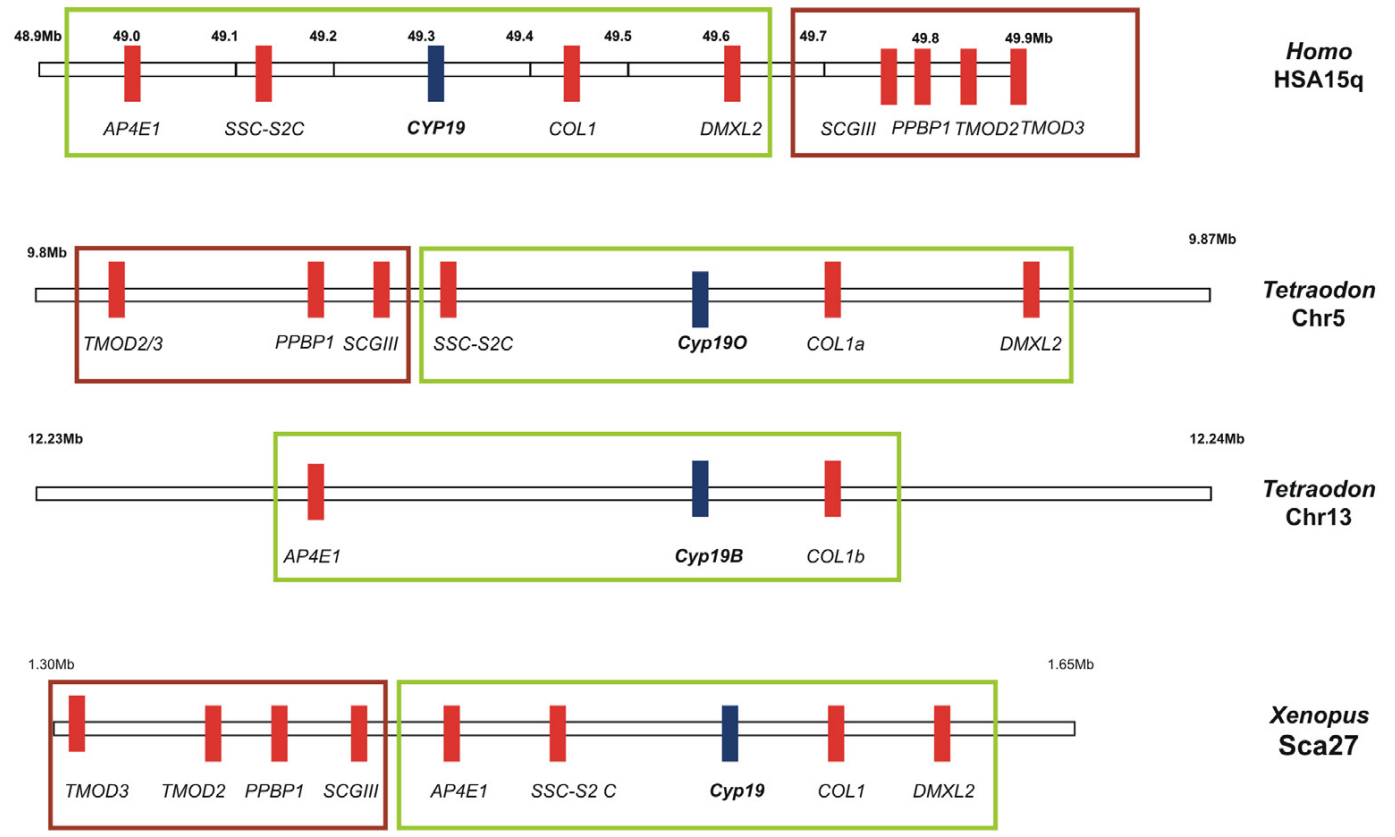
Physical maps of the genomic environment around *CYP19 *in *Homo sapiens*, *Tetraodon nigroviridis *and *Xenopus tropicalis*.

The human *CYP19 *maps to one of the proposed MHC-paralogon regions (HSA15q) (figure 1) [20]. Despite this proposal, no phylogenetic analysis has been performed to confirm that gene families at HSA15q are part of the MHC-paralogon. This strategy aimed at defining the presence/absence of invertebrate orthologues and the duplication timings of the selected gene families (both these questions are key predictions of paralogy regions). Therefore, we undertook the task of analysing the "genomic environment" surrounding the *CYP19 *human gene at HSA15q within a DNA sequence of 1.0 Mb (figure 2). Besides the *CYP19*, eight other ORFs are annotated within this DNA sequence, corresponding to the following genes: *AP4E1*, *FLJ41287*, *COL*, *DMXL2*, *SCGIII*, *MGC35274*, *TMOD2*, and *TMOD3 *(figure 2). This DNA module is outflanked by members of the Tropomodulin gene family, *TMOD2 *and *TMOD3*, which have been proposed to support the vertebrate genome duplication hypothesis [20]. Other members of the TMOD gene family map to expected regions of MHC paralogy (figure 1). Furthermore, a single orthologue is found in invertebrate species (e.g. *D. melanogaster *– *sanpodo*).

We began by investigating the gene complement for each gene family in vertebrate and invertebrate species through BLAST search. Phylogenetic analysis was then performed when no previous study was available to determine duplication timings (if duplication had occurred).

#### AP4-E1

**Figure 3:**
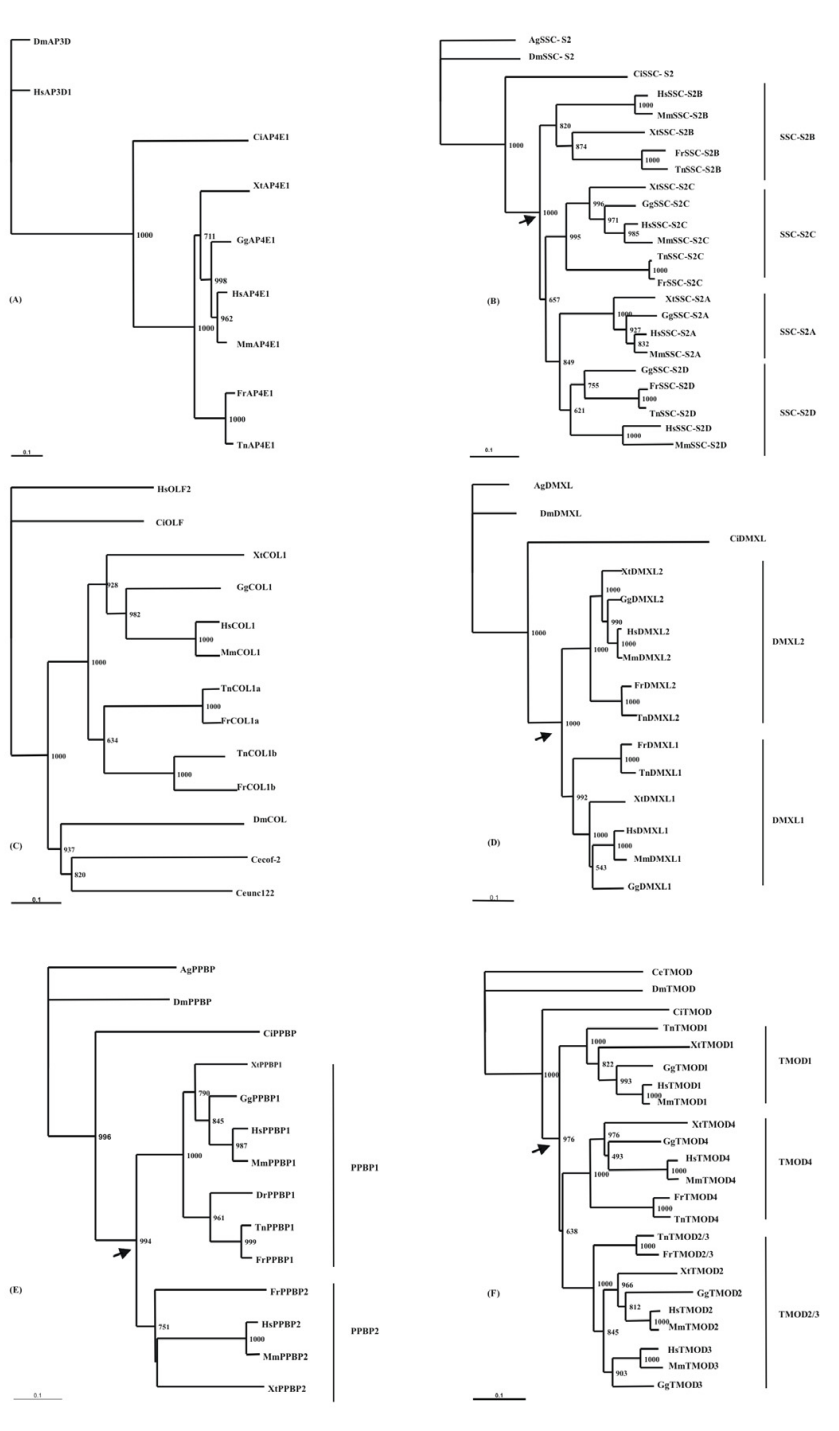
Neighbor-joining phylogenetic trees from alignment of the putative protein sequences of AP4E1 (A), SSC-2S (B), COL (C), DMXL (D), PPBP (E) and TMOD (F). Figures at nodes are scores from 1000 bootstrap resamplings of the data. Arrow denotes duplication timing. Ag – *Anopheles gambiae*; Ce – *Caenorhabditis elegans*; Dm – *Drosophila melanogaster*; Ci – *Ciona intestinalis*; Hs – *Homo sapeins*; Mm – *Mus musculus*; Gg – *Gallus gallus*; Xt – *Xenopus tropicalis*; Fr – *Fugu rubripes*; Tn – *Tetraodon nigroviridis*.

Adaptor protein complexes function as vesicle coat components in different membrane traffic pathways [24]. *AP4E1 *is a recently described subunit of a 4^th ^complex [24]. Up to the present day the *AP4E1 *gene has been found solely in vertebrate genomes. Through BLAST search we have found the first invertebrate sequence in the *C. intestinalis *genome (scaffold 11). As shown in the phylogenetic tree, *CiAP4E1 *is basal to the vertebrate genes with 1000 of bootstrap support (figure 3A). No further homologues were uncovered in vertebrate genomes.

#### SSC-S2

The ORF identified in Ensembl as NP_997264 (FLJ41287 protein), presents significant sequence similarity to three other GenBank entries. One of those is a novel tumor necrosis factor-α inducible gene, *SSC-S2 *[25], which maps to HSA5. *SSC-S2 *contains a motif in the amino terminus that shows a significant similarity to death effector domain II of cell death regulatory protein, Fas-associated death domain-like interleukin-1β-converting enzyme-inhibitory protein (FLIP) [25]. Through phylogenetic analysis we showed these four sequences to be paralogues (figure 3B). The four genes were named as follows: *SSC-S2A *(HSA5), *SSC-S2B *(HSA1), *SSC-S2C *(HSA15) and *SSC-S2D *(HSA19). Invertebrate orthologues were found in *Anopheles gambiae*, *D. melanogaster*, *C. elegans *(not used in the phylogeny) and *C. intestinalis *(scaffold 92). The duplication events date to early vertebrate origin, as indicated by the branching pattern of the tree (figure 3B). The invertebrate sequences are basal to the vertebrate genes with a significant bootstrap support (figure 3B). No homologue of *SSC-S2A *is found in Actinopterygii, possibly due to gene loss. Additionally, the human genes are all located in regions of MHC paralogy – HSA1, HSA19, HSA5 and HSA15 (figure 1), as expected from two rounds of "en bloc" duplication in early vertebrate ancestry. However, the tree branching pattern is not of the (A,B)(C,D) type, but sequential which is not in agreement with the "en bloc" scenario.

#### COL

The Ensembl annotation identifies this gene as Collomin. This as been renamed to Colmedin (*COL1*) [26]. Colmedin is a phylogenetically conserved type II transmembrane protein with collagen repeats and a cysteine-rich olfactomedin domain, with members described in *C. elegans *(two genes), *Drosophila *and vertebrates [26]. No orthologue was detected in *C. intestinalis*. Colmedin has been found to be a single-copy gene in several vertebrate species. BLAST search to *Danio *(not shown), *Fugu *and *Tetraodon *genomes uncovered a new Colmedin gene, which we name *COL1b *(figure 3C). *COL1b *is a specific paralogue of Actinopterygii. The genomic location of this new gene is explained most likely by an extra genome duplication (see following section).

#### DMXL

*RAB-3 *is a 12 WD domain protein which binds both GDP/GTP exchange protein and GTPase-activating protein for Rab3 small G protein family [27]. These domains are found in a variety of proteins and are likely to be involved in protein-protein interactions [28]. It shows a domain structure similar to that of *DMXL1 *which has 10 WD domains, and has been renamed *DMXL2 *[27,29]. Our phylogenetic analysis confirms that both genes are paralogues, with invertebrate sequences basal to vertebrate *DMXL1 *and *DMXL2 *(figure 3D). Moreover, *DMXL1 *maps to an expected region of MHC-paralogon in HSA5 (figure 1). Thus, the duplication event which originated *DMXL2 *and *DMXL1 *resulted most likely from two rounds of "en bloc" duplications in early vertebrate ancestry.

#### SCGIII

Secretogranin III (*SCGIII*) is a member of the granin protein family, that is a component of intracellular dense core vesicles. Through BLAST we found this gene family to be restricted to vertebrates (not shown).

#### NM_699205-PPBP

The ORF identified in Ensembl as NM_699205 codes for the hypothetical protein MGC35274. Sequence features (Lysin domain) indicate that it might be involved in cell wall catabolism. The *C. elegans *orthologue has been named Predicted peptidoglycan-binding protein (PPBP). Thus, we named the human gene *PPBP1*. A second PPBP gene can be found in vertebrate genomes, which we designate *PPBP2*. The phylogenetic tree indicates that both ORFs are paralogues (figure 3E). The second *PPBP *gene is present in *Fugu *(*Tetraodon *also has a second PPBP gene but due to the partial sequence was kept out of the phylogeny), amphibians and mammals. The tree pattern indicates that a duplication of an ancestral *PPBP *gene occurred specifically in the vertebrate lineage. Moreover, the second gene maps to an expected region of MHC paralogy in the human genome – HSA1.

#### TMOD2 and TMOD3

Popovici et al. [20] proposed that the TMOD gene family duplicated in the vertebrate lineage. However, no phylogenetic analysis was performed to support this assumption. In the human genome 4 tropomodulin genes have been annotated: *TMOD1 *(Erythrocyte tropomodulin), *TMOD4 *(Skeletal muscle tropomodulin), *TMOD2 *(Neuronal tropomodulin) and *TMOD3 *(Ubiquitous tropomodulin). In invertebrates a single tropomodulin gene is observed. The phylogenetic analysis by Almenar-Queralt et al. [30] suggests that *TMOD1*, *2 *and *4 *duplicated in the vertebrate lineage. Nevertheless, the origin of *TMOD3 *is still unclear. The genomic location of both *TMOD2 *and *3 *is highly suggestive for a tandem duplication. Our phylogenetic analysis, supports this scenario (*TMOD2 *and *3 *are tandem duplicates from an ancestral *TMOD2/3 *gene). The duplication event post-dates the divergence of fish and amphibians, since a single *TMOD2/3 *is found in both *Fugu *and *Tetraodon *(figure 3F). On the contrary, *Xenopus*, chicken, mouse and human have two distinct genes (mapping side by side) (the *Xenopus TMOD3 *orthologue has not been used in the phylogeny). Thus, we propose that a single tropomodulin gene existed in vertebrate ancestry. It duplicated to yield three TMOD genes (1, 2/3 and 4) as a result of "en bloc" duplication (probably as part of genome duplications). Later, a tandem duplication in the ancestor of *Xenopus*, chicken and mammals, originated the *TMOD2 *and *TMOD3 *genes.

The phylogenetic analysis of the full set of gene families within the human aromatase DNA segment, reveals that four of those have specifically duplicated in the vertebrate lineage. Only the SSC-2S gene family shows four paralogues. The tree branching pattern is not of the (A,B)(C,D) type (expected under an "en bloc" duplication) but sequential (expected under the adaptive duplication scenario). This observation has been interpreted as evidence against an "en bloc" scenario [31]. Thus, the phylogeny (branching patterns) *per se *does not support the "en bloc" duplications. In this context, the suggested duplicated regions could have resulted from a complex duplication, loss and rearrangement pattern, and not from "en bloc" duplications [18]. However, Furlong and Holland [23], have recently disputed this assumption.

Our analysis confirms the previous suggestion by Popovici et al. [20] that genes within HSA15q are part of the MHC-paralogon. We find that the paralogues for each gene family map to expected regions of MHC paralogy (figure 1). That is the case of *SSC-2S *(4 genes), *DMXL *(2 genes), *PPBP *(2 genes) and *TMOD *(3 genes). The physical proximity between these genes is also observed in other regions of paralogy besides HSA15. For example, paralogues *SSC-S2B*, *PPBP2 *and *TMOD4 *map closely in chromosome 1 (200 kb), while *DMXL1 *and *SSC-S2A *are separated by just 300 kb in chromosome 5 (figure 1). Furthermore, of those genes which are found to be single copy in vertebrates, only for *CYP19 *and *SCGIII *we have not found invertebrate orthologues (either in *C. intestinalis*, *C. elegans *or *D. melanogaster*).

### Comparative genomics

**Figure 4:**
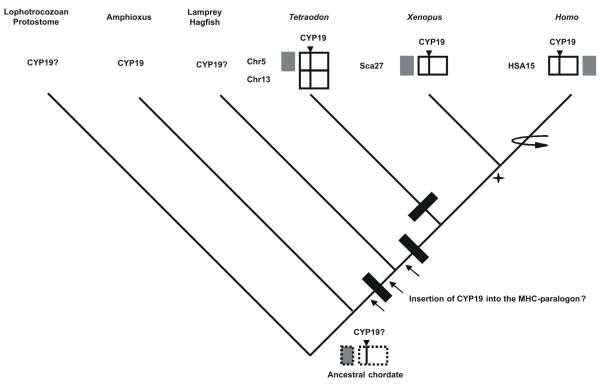
Evolutionary model for the origin of the aromatase gene family. Symbols from the two conserved DNA blocks from figure 2. Dotted line boxes denote conservation of synteny but not gene order. Black bars denote "en bloc" (or genome) duplications. Star indicates tandem duplication of TMOD2/3. Horizontal curve arrow indicates gene inversion of the DNA block containing TMOD3, TMOD2, PPBP and SCGIII.

Our phylogenetic analysis and paralogy study strongly suggests that the DNA segment harbouring the human aromatase gene resulted from an ancestral "en bloc" duplication of the MHC-paralogon. However, the question remains regarding the ancestry of human *CYP19 *genomic location. It could well be case that the human *CYP19 *location is of no relevant evolutionary meaning. In order to explore these possibilities, we compared the gene content and organisation around *CYP19 *genes in three vertebrate species: *H. sapiens*, *T. nigroviridis *and *X. tropicalis *(figure 2). The comparison indicates a striking pattern of conservation in both gene content and gene order in the three species (figure 2). No gene insertion occurred since the divergence of these lineages. Two genomic events took place: genomic inversion and tandem gene duplication (*TMOD2*, *TMOD3*). The DNA module containing *TMOD3*, *TMOD2*, *PPBP1 *and *SCGIII *is differently located in both fish/amphibian and humans (boxes figure 2). By comparing the gene order in the three clades it is possible to infer the ancestral configuration. Given that both *Tetraodon *and *Xenopus *have an identical gene order, it is more parsimonious to conclude that the *H. sapiens *configuration is derived (figure 4). The data indicates that prior to the divergence of these three lineages the aromatase gene was already at this precise location (figure 4). In the case of *Tetraodon*, this region has duplicated further onto chromosomes 5 and 13, originating *CYP19a *and *b*, most likely as the result of a further genome duplication on the teleost lineage (figure 4). Later in evolution, a genomic inversion has taken place in the human lineage (possible mammalian) (figure 4).

### CYP19 in amphioxus

**Figure 5:**
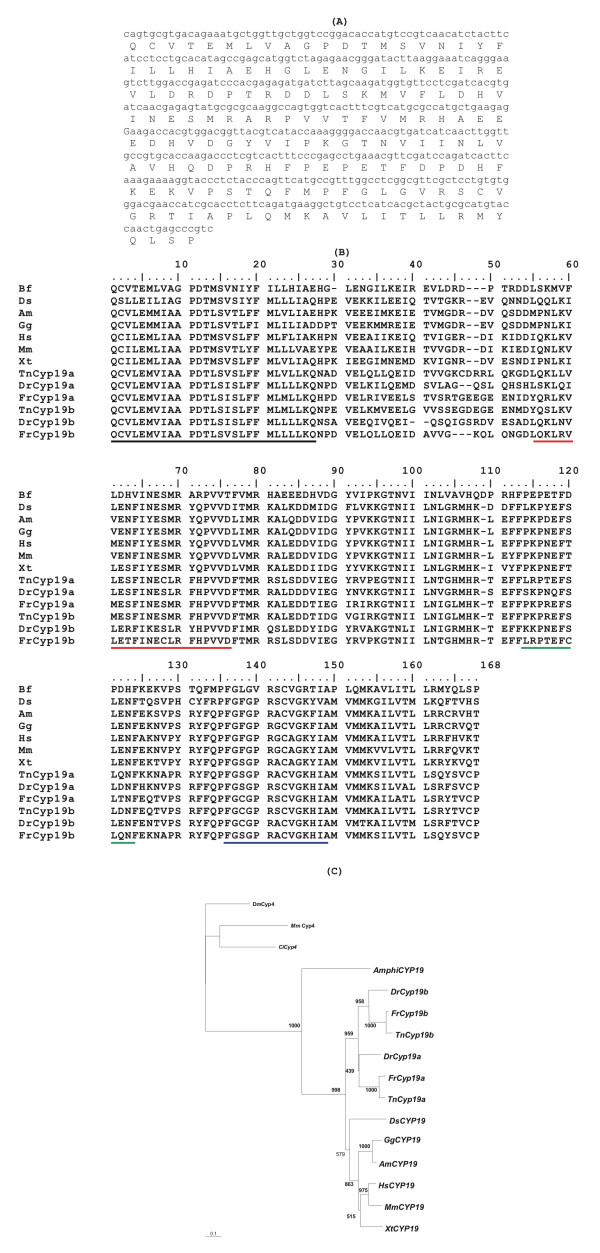
Nucleotide and predicted amino acid sequence of the *AmphiCYP19 *(partial sequence) **(A)**; alignment of CYP19 sequences; dashes denote insertions; black line, I-helix region; red line, Ozol's peptide region; green line, Aromatic region and blue line, Heme binding region. Bf, *B. floridae*, Ds, *D. sabina*, Am, *Alligator mississippiensis*, Gg, G. Gallus, Hs, *H. sapiens*, Mm, *M. musculus*, Xt, *X. tropicalis*, Tn, *T. nigroviridis*, Dr, *D. rerio*, Fr, *Fugu rubripes ***(B)**; Neighbor-joining phylogenetic tree from the alignment of the putative protein sequences of *CYP19 *genes, figures at nodes are scores from 1000 bootstrap resamplings of the data ; an insertion of the *TnCYP19b *predicted protein sequence was kept out of the alignment **(C)**.

The phylogenetic analysis, paralogy study and the conservation of gene organisation around aromatase in three vertebrate species suggest that *CYP19 *(and the immediate outliers) was present in the invertebrate unduplicated MHC-paralogon prior to vertebrate radiation. However, they do not rule out the possibility that *CYP19 *emerged either just before or after the divergence of lamprey/hagfish and gnathostomes (being inserted into the MHC paralogy regions) (arrows-figure 4). The exact timing of the proposed two rounds of genome duplications which structure the vertebrate genome is still contentious. However, the consensus points to one duplication prior to the divergence of lamprey/hagfish and gnathostomes, and the second after the divergence of these lineages [32] (figure 4). Furthermore, in the genome sequence of the sea squirt despite the evidence of an MHC unduplicated paralogon [33], no orthologue of aromatase has been found [10]. On the contrary, amphioxus has proved a more favourable model to address these issues [23]. In order to investigate the presence of *CYP19 *in *B. floridae*, a BLAST search to the trace archives of the Whole Genome Sequence using the *CYP19 *sequence from the stingray (*Dasyatis sabina*) was performed. A single hit with a significant *E*-value was retrieved. This information was subsequently used to isolate a partial sequence (494 bp) from DNA extracted from a cDNA 5–24 h embryo library. In figure 5A, we show the DNA sequence and the predicted amino acid translation. When run on BLAST, it clearly emerges that it belongs to the CYP19 gene family. We designate this *AmphiCYP19*. Vertebrate aromatase genes display typical putative structural domains. These include the I-helix region, Ozol's peptide region, Aromatic region and Heme-binding region [34] (figure 5B). The alignment provided in figure 5B indicates the presence of similar motifs in the *AmphiCYP19 *sequence we now describe. The orthology of the retrieved sequence was determined through phylogeny. Overall, this analysis strongly indicates that *AmphCYP19 *is part of the aromatase evolutionary clade (bootstrap of 1000) (figure 5C). Our result suggests that that a single *CYP19 *gene was present at the base of the chordate lineage. We propose that it has been independently lost in the urochordate *C. intestinalis*. We argue that the ancestral invertebrate chordate *CYP19 *gene underwent two rounds of "en bloc" duplication like many other gene families in the MHC-paralogon to yield four paralogues. Three of these have been lost. However, we cannot rule out that no CYP19 duplications occurred in the vertebrate lineage, regardless of whether "en bloc" duplications occurred in other gene families. We favour the first scenario.

The present results imply a significant theoretical change regarding the ancestry of the CYP19 gene family. This investigation started with the observation that the human aromatase gene maps to the MHC-paralogon. Nevertheless, two opposite scenarios could be draw from the phylogenetic analysis, paralogy and comparative genomics. Either the *CYP19 *locus was present in the invertebrate chordate unduplicated MHC-paralogon, and the presence of a single paralogue resulted from gene loss; *or *CYP19 originated early on in vertebrate evolution in its present position in the MHC-paralogon (figure 4). We went on to test these hypotheses. Our results can be summarised as follows: **(1) **vertebrate CYP19 containing regions are indeed part of the MHC-paralogon as demonstrated by the phylogenetic analysis of the gene families in close proximity; **(2) **comparative genomics of the aromatase region between fish, amphibians and humans shows a striking pattern of conservation without any gene insertion; **(3) **following the previous analysis, we found that *CYP19 *is not restricted to the vertebrate clade, given the description of *AmphiCYP19*.

Our model determine the loss of three aromatase paralogues upon duplication of the ancestral MHC-paralogon. For the vast majority of paralogy regions it is difficult to precisely determine the amount of gene loss (due to the absence of large sets mapping data from crucial organisms). In the case of the MHC-paralogon an estimate can be calculated, given the previous work of several authors [17,21,35]. The sequencing and mapping data of the MHC anchor genes in amphioxus, shows a significant proportion of gene families which are single-copy in both lineages (seven out of eighteen, excluding the anchor genes and those of unknown orthology; e.g. frequenin-like) [17,35]. Thus, the return to a single copy status following the "en bloc" duplication was not a rare event upon the duplication of the MHC-paralogon. At the moment we do not known whether *AmphiCYP1*9 maps along with the MHC anchor genes in a single chromosome, but this hypothesis can be tested in the future [36].

Finally, we speculate that the ancestry of *CYP19 *genes could be more ancient than invertebrate chordate origin. Two reasons support this scenario. First, the sex steroid receptors (estrogens and androgens/progesterone/corticoids) are older than previously proposed [13]. The estrogen receptor found in *Aplysia *indicates that the duplication event from a sex steroid precursor receptor pre-dates the divergence of protostomes and deuterostomes [13]. Also, the phylogenetic analysis and paralogy studies of androgen, progesterone and corticoid receptors suggests that a single receptor was present in the ancestral Urbilateria [37]. Thus, the receptor gene kit for sex steroid hormones was already present in the primitive Bilateria (albeit not necessarily with a similar function). The second reason comes from Lophotrocozoan molluscs. These organisms respond to steroid hormones (e.g. estradiol) during their reproductive cycle [38]. Furthermore, biochemical analysis in mollusc tissue extracts reveals the presence of an aromatase-like activity [39,40]. In light of these findings and observations, we argue that the presence of *CYP19 *should be investigated in lophotrocozoan protostomes (e.g. molluscs).

## Conclusion

We present here a detailed study of the genomic region containing the aromatase gene in three vertebrate lineages. The gene families found in close proximity to *CYP19 *show a clear pattern of vertebrate specific duplication, as expected from a paralogon. A key prediction from paralogy regions is their unduplicated presence in pre-vertebrate genomes. Significantly, we have also found that the genomic organisation of the human *CYP19 *genomic region mimics that of *Tetraodon *and *Xenopus*. Overall our analysis suggested the existence of aromatase in invertebrates. In agreement with this hypothesis we have found a *CYP19 *orthologue in the invertebrate chordate amphioxus. Contrary to previous suggestions, our data implies that *CYP19 *was present in the primitive chordate (and probably even earlier).

## Methods

### Phylogenetics and paralogy studies

The gene content around the *CYP19 *human gene in chromosome 15 comprises eight open reading frames (ORFs) within a DNA sequence of 1.0 Mb (figure 2). These are annotated as follows: *AP4-E1 *[GenBank: NP_031373], *FLJ41287 *(SSC-2SC) [GenBank: NP_997264], *Collomin *[GenBank: NP_861454], *DMXL2 *[GenBank: NP_056078], *SCGIII *[GenBank: NP_037375], *MGC35274 *[GenBank: NP_699205], *TMOD2 *[GenBank: NP_055363], and *TMOD3 *[GenBank: NP_055362] (figure 2). In order to find invertebrate orthologues and vertebrate paralogues, protein sequence from each gene was extracted and used for BLAST search (TBLASTN) against GenBank and Ensembl. Accession numbers for each gene are given in table 1.

**Table 1:** Accession numbers for the genes used in the phylogenetic analysis.

**Gene family**	**Gene**	**Accession number**	**Species**
**AP4E1**		NP_031373	*H. sapiens*
		ENSMUSP00000002063	*Mus musculus*
		ENSGALP00000007610	*Gallus gallus*
		SINFRUP00000132389	*Fugu rubripes*
		GSTENG00025592001	*T. nigroviridis*
		ENSXETP00000044864	*X. tropicalis*
		ci0100133154** (scaffold11)**	*C. intestinalis*
**SSC-S2**		ENSANGP00000018260	*A. gambiae*
	CG4091	AAF47048	*D. melanogaster*
		ci0100140958** (scaffold 92)**	*C. intestinalis*
	**A**	NP_055165	*H. sapiens*
		ENSMUSP00000034810	*M. musculus*
		ENSGALP00000007631	*G. gallus*
		ENSXETP00000044865	*X. tropicalis*
	**B**	NP_078851	*H. sapiens*
		NP_081482	*M. musculus*
		ENSXETP00000017843	*X. tropicalis*
		SINFRUP00000149825	*F. rubripes*
		GSTENT00011674001	*T. nigroviridis*
	**C**	NP_997264	*H. sapiens*
		ENSMUSP00000034810	*M. musculus*
		ENSGALP00000007631	*G. gallus*
		ENSXETP00000044865	*X. tropicalis*
		SINFRUT00000165316** annotated as pseudogene**	*F. rubripes*
		GSTENT00018057001	*T. nigroviridis*
	**D**	NP_689575	*H. sapiens*
		ENSMUSP00000076961	*M. musculus*
		ENSGALP00000006772	*G. gallus*
		SINFRUP00000156999	*F. rubripes*
		GSTENT00028868001	*T. nigroviridis*
**Colmedin**	CG6867	NP_573262	*D. melanogaster*
	COF-2	AY494975	*C. elegans*
	unc-122	AY494976	*C. elegans*
	**COL1**	NP_861454	*H. sapiens*
		NP_796324	*M. musculus*
		ENSGALP00000021668	*G. gallus*
		ENSXETP00000044876	*X. tropicalis*
	**COL1a**	SINFRUP00000165325	*F. rubripes*
		GSTENG00018059001	*T. nigroviridis*
	**COL1b**	SINFRUP00000132381	*F. rubripes*
		GSTENT00025590001	*T. nigroviridis*
**DMXL**		XP_314464.1	*A. gambiae*
	DmX	NP_572302	*D. melanogaster*
		CAB01916	*C. elegans*
		ci0100136505** (scaffold149)**	*C. intestinalis*
	**DMXL2**	ENSGALP00000007662	*G. gallus*
		NP_056078.1	*H. sapiens*
		XP_358382.2	*M. musculus*
		GSTENG00018060001	*T. nigroviridis*
		SINFRUP00000166673	*F. rubripes*
		ENSXETP00000044880	*X. tropicalis*
	**DMXL1**	ENSGALP00000003481	*G. gallus*
		CAA06718	*H. sapiens*
		ENSMUSP00000045559	*M. musculus*
		GSTENG00030946001	*T. nigroviridis*
		SINFRUP00000162769	*F. rubripes*
		ENSXETP00000038136	*X. tropicalis*
**PPBP**	**PPBP1**	NP_699205	*H. sapiens*
		GSTENG00018055001	*T. nigroviridis*
		SINFRUP00000165314	*F. rubripes*
		ENSGALP00000007561	*G. gallus*
		NP_081585	*M. musculus*
	**PPBP2**	CAI16380	*H. sapiens*
		ENSMUSP00000067811	*M. musculus*
		ENSXETP00000017826	*X. tropicalis*
		SINFRUP00000149824	*F. rubripes*
		ENSXETP00000044860	*X. tropicalis*
		ci0100137161** (scaffold34)**	*C. intestinalis*
		XP_321699	*A. gambiae*
		NP_650352	*D. melanogaster*
**TMOD**	**TMOD**	AAL13319	*C. elegans*
	**TMOD (*spdo*)**	AAC04506	*D. melanogaster*
	**TMOD**	ci0100140287** (scaffold487)**	*C. intestinalis*
	**TMOD1**	NP_003266	*H. sapiens*
		NP_068683	*M. musculus*
		NP_990105	*G. gallus*
		ENSXETP00000022442	*X. tropicalis*
		GSTENG00033528001	*T. nigroviridis*
		SINFRUP00000157299	*F. rubripes*
	**TMOD2/3**	GSTENG00018054001	*T. nigroviridis*
		SINFRUP00000165312	*F. rubripes*
	**TMOD2**	NP_055363	*H. sapiens*
		Q9JKK7	*M. musculus*
		ENSGALP00000007536	*G. gallus*
		ENSXETP00000044853	*X. tropicalis*
	**TMOD3**	NP_055362	*H. sapiens*
		ENSMUSP00000072087	*M. musculus*
		ENSGALP00000007492	*G. gallus*
		ENSXETP00000044845	*X. tropicalis*
	**TMOD4**	NP_037485	*H. sapiens*
		NP_057921	*M. musculus*
		NP_990105	*G. gallus*
		SINFRUP00000149822	*F. rubripes*
		GSTENG00011677001	*T. nigroviridis*
		ENSXETP00000044853	*X. tropicalis*
**CYP19**		NP_112503	*H. sapiens*
			*B. floridae*
		AAF04617	*D. sabina*
		AAK31803	*Alligator mississippiensis*
		NP_001001761	*G. gallus*
		P28649	*M. musculus*
		ENSXETP00000044866	*X. tropicalis*
	**Cyp19a**	GSTENP00025591001	*T. nigroviridis*
	**Cyp19b**	GSTENP00018058001	*T. nigroviridis*
	**Cyp19a**	NP_571229	*D. rerio*
	**Cyp19b**	NP_571717	*D. rerio*
	**Cyp19a**	SINFRUP00000132385	*F. rubripes*
	**Cyp19b**	SINFRUP00000165318	*F. rubripes*
			
**Others**	**AP3D**	AAC14585	*D. melanogaster*
	**AP3D1**	NP_003929	*H. sapiens*
	**OLFM2**	ENSP00000264833	*H. sapiens*
	**CiOLF**	ci0100131069**(scaffold4)**	*C. intestinalis*
	**Cyp4d2**	Z23005	*D. melanogaster*
	**CYP4**	ENSMUSP00000003574	*M. musculus*
	**CYP4**	ci0100146084** (scaffold15)**	*C. intestinalis*

Putative protein sequences for each gene family were aligned using the CLUSTAL X program (version 1.8). The produced alignments were further edited by eye to maximise the homologous regions (conserved domains) (given upon request). The phylogenetic reconstruction was based on conserved domains. If no domains were identified, reconstruction's were performed using the full-length alignment (without taking into account gaps or ambiguous sites). The phylogenetic trees were constructed using neighbor-joining from the CLUSTAL X program, on an amino acid distance matrix calculated with the Dayoff PAM option. Confidence on each node was assessed by 1000 bootstrap replicates. Trees were visualised with the Treeview program (version 1.6.6).

Mapping data was retrieved from *H. sapiens *[41], *T. nigroviridis *[42], *X. tropicalis *[43] and *C. intestinalis *[44].

### Polymerase chain reaction (PCR)

A BLAST search to the *B. floridae *trace archives of the Whole Genome Sequence using the DNA sequence from the stingray (*D. sabina*) *CYP19 *was done. Clone AFSA830540 presented a significant *E*-value (data not shown). Clone walk 5' and 3' allowed the determination of further regions of *CYP19 *homology. To obtain a *CYP19 *sequence fragment an hemi-nested PCR approach was followed using DNA purified from an 5–24 h embryo cDNA library (J. Langeland, Kalamazoo, USA). Three oligonucleotides (2 forward and 1 reverse) were designed in conserved regions using the available genomic sequence: CYPF1 5' CTGGCTAACATCCGGGACAT 3'; CYPF2 5' CAGTGCGTGACAGAAATGCT 3'; and CYPR1 5' GACGGGCTCAGTTGGTACAT 3'. PCR was carried out in 50 μl reaction mixture consisting of 10 mM Tris-HCl, pH 8.0, 1.5 mMMgCl_2_, KCl 50 mM, TritonX 0.1%, 10 μM of each primer, 2 mM each of dATP, dCTP, dGTP, and dTTP, 1U DNA polymerase (Appligene Oncor). The first round of PCR (oligonucleotides CYPF1 and CYPR1) had the following profile: initial cycle of denaturation, 94°C 2 min, and forty amplification cycles with denaturation at 94°C for 45 s, annealing at 55°C for 30 s, and extension at 72°C for 1 min. An hemi-nested PCR was carried out afterwards using the first PCR product as a sample. A similar PCR profile was used with the exception of the extension time – 45 s (oligonucleotides CYPF2 and CYPR1). The PCR product was separated through 2% agarose gel and purified by using the QIAquick Gel Extraction kit (QIAGEN, Germany). The product was directly sequenced in both strands using the PCR oligonucleotides by STAB VIDA (Portugal). The sequence was deposited in Genbank DQ085624.

## Authors' contributions

LFCC performed all sequence and phylogenetic analysis, comparative genomics, laboratory experiments and drafted the manuscript, MMS participated in phylogenetic analysis, design and co-ordination of the study, and MARH participated in the co-ordination of the study.
